# *Mycobacterium conceptionense* Infection after Breast Implant Surgery, France

**DOI:** 10.3201/eid1607.090771

**Published:** 2010-07

**Authors:** Sandrine Thibeaut, Pierre-Yves Levy, Marie-Laure Pelletier, Michel Drancourt

**Affiliations:** Author affiliations: Université de la Méditerranée, Marseille, France (S. Thibeaut, P.-Y. Levy, M. Drancourt);; Clinique la Casamance, Aubagne, France (P.-Y. Levy, M.-L. Pelletier)

**Keywords:** Bacteria, Mycobacterium conceptionense, tuberculosis and other mycobacteria, nontuberculous mycobacteria, infection, breast implant complications, Mycobacterium fortuitum, post surgical infection, letter, *Suggested citation for this article:* Thibeaut S, Levy P-L, Pelletier M-L, Drancourt D. *Mycobacterium conceptionense* infection after breast implant surgery, France [letter]. Emerg Infect Dis [serial on the Internet]. 2010 Jul [*date cited*]. http://dx.doi.org/10.3201/eid1607.090771

**To the Editor**: *Mycobacterium fortuitum* complex members are rapidly growing mycobacteria found in water and soil (*1*). These opportunistic pathogens are responsible for posttraumatic skin and soft tissue infections. They also account for 60%–80% of postsurgical wound infections caused by rapidly growing mycobacteria (*2*), particularly after breast surgery (with or without prosthetic implants) (*3*). *M. conceptionense*, an emerging member of the *M. fortuitum* complex, was initially described in a case of osteomyelitis that occurred after an open fracture of the tibia (*4*). We report a case of *M. conceptionense* infection that occurred after breast surgery.

A woman 58 years of age had a left mastectomy with lymph node dissection and chemotherapy for breast carcinoma in March 2004. Three years later, she underwent breast reconstruction that used a cutaneomuscular latissimus dorsi flap with a prosthetic implant. Immediately after surgery, a fever (39°C) developed, but 3 blood cultures remained sterile. No treatment was administered, and she became afebrile within 3 days.

At day 15 after surgery, a serous discharge appeared in the tip of the skin flap. By day 21, the patient was again febrile, and the wound discharge was swabbed for analysis. On day 27, she underwent surgical revision with ablation of the breast implant, drainage, and sample collection. The leukocyte count was normal. However, the C-reactive protein level was 99 mg/L, and the erythrocyte sedimentation rate was 111 mm (first hour). Treatment with intravenous amoxicillin/clavulanic acid was started. Although the biologic parameters normalized, the serous discharge continued. Microscopic examination of specimens from days 21 and 27 yielded no bacteria in Gram- and Ziehl-Nielsen–stained pus specimens, and standard bacteriologic cultures remained sterile. *M. conceptionense*, identified by partial *rpo*B gene sequencing (100% identity with GenBank accession no. AY859695.1) (*4*), grew in both specimens after 8 days of incubation at 37°C under a 5% CO_2_ atmosphere in Coletsos medium (bioMérieux, La Balme-les-Grottes, France). By the Etest method (*4*), both isolates were susceptible to several antimicrobial drugs, including clarithromycin, amikacin, ciprofloxacin, and doxycycline. The patient was treated with ciprofloxacin, azythromycin, and amikacin for 3 weeks, followed by ciprofloxacin and azythromycin for 4 weeks.

At patient’s relapse 3 months later, *M. conceptionense* exhibiting identical antimicrobial drug susceptibility pattern was again isolated from the wound fluid. The patient was then treated with ciprofloxacin, azythromycin, and doxycycline for 6 months; subsequently, doxycycline alone was given for a total of 18 months. Results from the 2-month follow-up examination were unremarkable.

*M. conceptionense* was unambiguously identified by partial *rpoB* gene sequencing, a first-line tool for accurate identification of nontuberculous mycobacteria (*5*). A pathogenic role for *M. conceptionense* was supported by 1) its repetitive isolation from the wound; 2) the absence of any other pathogen; and 3) wound healing during appropriate, long-term antimicrobial drug treatment. However, the source of infection remained unknown. The patient had a tattoo on the skin flap used for the breast reconstruction, and ink has been shown to be a source for rapidly growing mycobacteria other than *M. conceptionense* (*6*). However, the tattoo was 5 years old and clinically safe.

Recent reports have identified 12 *M. conceptionense* isolates from water collected in a cooling tower (*7*) and 9 isolates from various freshwater fish species (*8*). The type strain of *M. conceptionense* had been isolated after prolonged exposure of the patient to fresh water (*4*). These observations suggest that water is a potential source for *M. conceptionense*. In this patient, use of contaminated aqueous solutions or inadequately rinsed surgical equipment (*9*) was unlikely the source of infection because further investigations indicated proper use of sterilized, nonreusable surgical equipment. At home, the patient used well water, but results of tests used to detect *M. conceptionense* by culture and the presence of the *rpoB* gene in well water remained negative.

Because *M. conceptionense* is an emerging pathogen with only 2 reported cases of infection (*4**,**10*), the optimal treatment is unknown (Table). Current recommendations for breast implant infection from *M. fortuitum* include 6 months of appropriate antimicrobial drug treatment in addition to implant removal because surgery alone yields relapse within 4–6 weeks (*2**,**3*). Increased worldwide use of breast implants is likely to increase the number of *M. conceptionense* infections and will raise questions about the optimal management of these infections.

**Table:** Three cases of *Mycobacterium conceptionense* infection in female patients*

Patient age, y	Clinical situation	Identification	Treatment		Reference
			Nature	Duration, mo	
31	Posttraumatic osteitis	16S rRNA, *soda, hsp65*, *recA*, *rpoB*†	Antimicrobial drug therapy: AMC	3	(*4*)
43	Subcutaneous abscess without trauma	partial 1,464-bp 16S rRNA gene‡	Surgery and antimicrobial drug therapy: COT and CLA; then DOX and CLA; then LIN and CLA	5	(*10*)
58	Breast implant infection	*rpo*B§	Surgery and antimicrobial drug therapy: CIP and AZY; then CIP, AZY, and DOX; then DOX	18	This report

*AMC, amoxicillin/clavulanic acid; COT, cotrimoxazole; CLA, clarithromycin; DOX, doxycycline; LIN, linezolid; CIP, ciprofloxacin, AZY, azythromycin. The outcome for all 3 patients was favorable. †GenBank accession nos.: 16S rRNA, AY859684; *rpoB*, AY859695; *hsp65*, AY859678; *sodA*, AY859708; *recA*, AY859690. ‡GenBank accession no. AM884289.1. §GenBank accession no. AY859695.1.
